# Comparative Study of Potential Habitats for Two Endemic Grassland Caterpillars on the Qinghai-Tibet Plateau Based on BIOMOD2 and Land Use Data

**DOI:** 10.3390/insects15100781

**Published:** 2024-10-08

**Authors:** Chuanji Li, Yunxiang Liu, Youpeng Lai, Hainan Shao

**Affiliations:** 1 State Key Laboratory of Plateau Ecology and Agriculture, Academy of Agricultural and Forestry Sciences, Qinghai University, Xining 810016, China; lichuanji0103@163.com (C.L.); 17791394452@163.com (Y.L.); yplai@126.com (Y.L.); 2 Provincial Key Laboratory of Agricultural Integrated Pest Management in Qinghai, Academy of Agricultural and Forestry Sciences, Qinghai University, Xining 810016, China

**Keywords:** *Gynaephora*, ensemble model, spatial distribution, ecological factors, alpine meadow protection, glacial period refuges

## Abstract

**Simple Summary:**

Grassland caterpillars in the Qinghai-Tibet Plateau (QTP) region are notorious for inflicting severe damage to the alpine meadow ecosystem. Currently, scarce research has been conducted on the spatial distribution patterns and potential habitats for these insect species. A significant difference between the potential habitats of *Gynaephora menyuanensis* and *G. qinghaiensis* is reported. The areas of their suitable habitats are significantly and positively correlated with the area of grassland among all land use types of QTP. The potential habitats of both species during the paleoclimate period were located in the eastern and southeastern boundary areas of the QTP. During the paleoclimate period, their potential habitats expanded towards the Hengduan Mountains (low-latitude regions) in the south compared with their current period suitable habitats. With the subsequent temperature rising, their distribution centers were shifted towards the northeast (high-latitude) regions. This study provides an important reference for designating the prevention and control areas for *Gm* and *Gq*.

**Abstract:**

This study has systematically investigated and compared the geographical distribution patterns and population density of *G. menyuanensis* (*Gm*) and *G. qinghaiensis* (*Gq*), which are endemic to the QTP region and inflict severe damage. Using a method combining the BIOMOD2 integration model (incorporating nine ecological niche models) and current species distribution data, this study has compared changes in potential habitats and distribution centers of these two species during ancient, present, and future climate periods and conducted a correlation test on the prediction results with land use types. The study results indicate that there are differences in geographical distribution patterns, distribution elevations, and population density of these two species. Compared with single models, the integration model exhibits prominent accuracy and stability with higher KAPPA, TSS, and AUC values. The distribution of suitable habitats for these two species is significantly affected by climatic temperature and precipitation. There is a significant difference between the potential habitats of these two species. *Gm* and *Gq* are distributed in the northeastern boundary area and the central and eastern areas of the QTP, respectively. The areas of their suitable habitats are significantly and positively correlated with the area of grassland among all land use types of QTP, with no correlations with the areas of other land use types of QTP. The potential habitats of both species during the paleoclimate period were located in the eastern and southeastern boundary areas of the QTP. During the paleoclimate period, their potential habitats expanded towards the Hengduan Mountains (low-latitude regions) in the south compared with their current suitable habitats. With the subsequent temperature rising, their distribution centers shifted towards the northeast (high-latitude) regions, which could validate the hypothesis that the Hengduan Mountains were refuges for these species during the glacial period. In the future, there will be more potential suitable habitats for these two species in the QTP. This study elucidates the ecological factors affecting the current distribution of these grass caterpillars, provides an important reference for designating the prevention and control areas for *Gm* and *Gq*, and helps protect the alpine meadow ecosystem in the region.

## 1. Introduction

The Qinghai-Tibet Plateau (QTP), which is renowned as the “Water Tower of China”, is a source area of many important rivers in Asia. With its unique geographical location and topographical features, it plays an important role in the global climate system and ecological environment [1,2]. The Qinghai-Tibet Plateau primarily involves the ecosystems of alpine grassland, wetland, and desert [3]. These ecosystems play a crucial role in maintaining climate stability and biodiversity [1,2]. However, ecosystems in this region are extremely fragile. In particular, the grassland ecosystem is very vulnerable to the threats of external factors (such as climate change, overgrazing, and pest outbreaks), which can lead to ecological issues, including soil erosion [4,5,6]. Frequent occurrences of extreme climate events and variations in precipitation patterns on the Qinghai-Tibet Plateau pose unprecedented challenges to the grassland ecosystem in the region [7]. These climate changes can impact the distribution patterns of potential habitats of insect pest species on the Qinghai-Tibet Plateau (such as *Gryllotalpa orientalis* and *Gypsonoma minutana*) [8]. They could adapt to these changes through phenotypic plasticity, genetic adaptation, or change in potential distribution ranges [9,10,11]. Therefore, understanding their dynamics under climate change is conducive to coping with the potential risks of biological disasters caused by climate change in the future [12,13,14,15].

During recent decades, with the protection of the ecological environment and the continuous rising of climate temperature, the varieties and quantities of plants in the grassland ecosystem have gradually increased [16]. This has provided abundant food sources for herbivorous insects, whose varieties and quantities have consequently increased [16,17]. Outbreaks of grassland pests result in the overconsumption of host plants, thus further impacting the health and stability of the entire grassland ecosystem [18]. Extreme climate events caused by the continuous rising of temperature can also affect the ecological niches and population dynamics of herbivorous insects and alter their life cycles and reproductive patterns. Therefore, the structure and functions of the entire grassland ecosystem can be affected through food chains and ecological interactions [1]. These adverse climate factors impact the activity of predators and parasites, thus mitigating the natural control effect of herbivorous insect populations [19]. Investigating the adaptability and distribution pattern variations of herbivorous insects to climate changes in the grassland ecosystem is of important significance for developing effective pest management strategies in the ecosystem and implementing proactive response strategies to mitigate the adverse impacts of these changes [19].

With human population growth and economic development, there are land conversions to agricultural and grassland, which in turn lead to increasing grazing and farming activities that further reduce the natural recovery capacities of ecosystems [20]. Changes in land use types on the Qinghai-Tibet Plateau have also significantly impacted its grassland ecosystem. Against this backdrop, the negative pressure of grassland pests on the grassland ecosystem combined with human activities has posed serious challenges to ecological conservation in the QTP region [19]. Grassland caterpillars are among the most harmful pests in the alpine meadow ecosystem of the QTP [21,22,23,24]. There are a total of fifteen grassland caterpillars in the world, which are primarily distributed in the alpine and Arctic regions of the Northern Hemisphere, with eight endemic species (*Gynaephora alpherakii*, *G. aureate, G. groenlandica*, *G. jiuzhiensis*, *G. menyuanensis*, *G. minora*, *G. qinghaiensis*, and *G. qumalaiensis*) in the QTP region [21,22,25,26,27]. *Gynaephora menyuanensis* and *G. qinghaiensis* are two closely related caterpillars that are most studied and widely distributed with the highest harmful levels [22,28,29,30,31,32]. Their larvae primarily feed on Poaceae and Cyperaceae plants in alpine meadows with significant consumption amounts [33]. Therefore, these pests have reduced the vegetation coverage rate of grassland and caused soil erosion and ecological function degradation, posing a serious threat to the stability of the grassland ecosystem in the QTP region [34,35]. The life history and ecological adaptability of these two pests demonstrate their high adaptability to alpine environments [22], indicating that these pests can complete their life cycles in low-temperature environments with little oxygen. At the same time, their capabilities of large-scale reproduction and dispersal ensure their rapidly increased population sizes under extreme environmental conditions, posing very extensive damaging ranges and extreme difficulties for prevention and control efforts. Therefore, these pests can wreak havoc on the grassland ecosystems [36,37]. Currently, scarce research has been conducted on the spatial distribution patterns of these two pests and their potential habitats. Under such circumstances, a comparative study of their species distribution patterns can facilitate the understanding of occurrences of these pest disasters and the development of effective prevention and control areas.

Species distribution models (SDMs) and ecological niche models (ENMs) are commonly used tools for studying how macro-ecological processes drive species distribution patterns and dynamics. These models (GARP and MAXENT) have become core tools for exploring species distribution patterns, habitat suitability, and potential driving factors, playing a key role in the biogeographic theory and applied research [38]. Currently, single ecological models can independently predict the potential distribution areas of species. However, these models present certain preferences and shortcomings, making it difficult to achieve accurate prediction results [39]. In particular, these models have many limitations in assessing the influences of environmental factors such as climate change and land use on species distribution patterns and identifying potential habitats of species to establish effective species management [38,39,40]. Therefore, integration models were developed to overcome the limitations of single models. These integration models can improve prediction accuracy by integrating multiple different prediction models [41]. Among these models, the BIOMOD2 model presents an excellent example that can be run simultaneously with ten different model parameters (including MAXENT, GLM, GAM, etc.) [42].

Therefore, this study comprehensively used the data of field surveys, environmental factor analyses, and land use, as well as the BIOMOD2 integration model, to conduct a comparative prediction of the potential habitats of these two species and verified the following three hypotheses: (1) Given their similar morphological characteristics, host preferences, and life history characteristics, the spatial distribution patterns of these two closely related pest species could be impacted by identical ecological factors and land use types. (2) Distribution patterns of suitable habitats of grassland caterpillars in high-altitude areas are more susceptible to temperature changes than those of caterpillars in low-altitude areas. (3) The Hengduan Mountains provided shelter for multiple species during the glacial period.

## 2. Materials and Methods

### 2.1. Study Area Overview, Species Identification, and Distribution Data Collection

**Figure 1:**
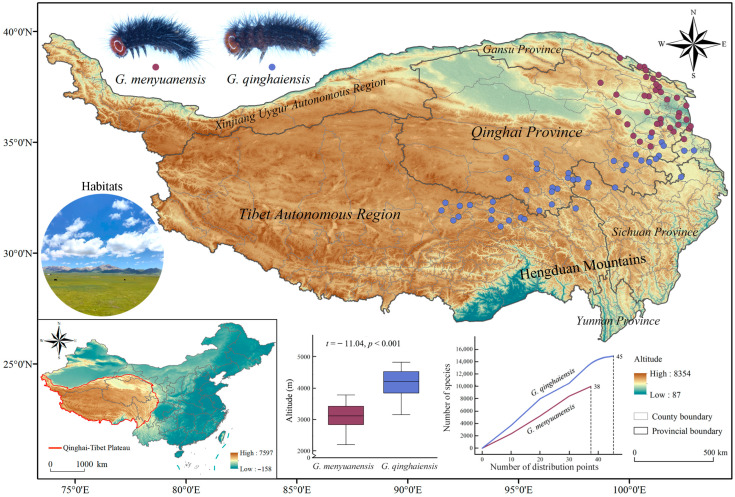
Geographic location of the Qinghai-Tibet Plateau and distribution points of *Gm* and *Gq.* Habitat graphics: typical habitats of *Gm* and *Gq*. Box plot: altitude distribution of *Gm* and *Gq*. Line chart: species accumulation curves of *Gm* and *Gq*.

From March 2020 to August 2023, field surveys were conducted, and larval species of *G. menyuanensis* (*Gm*) and *G. qinghaiensis* (*Gq*) were collected on the Qinghai-Tibet Plateau (26°00′12″ N~39°46′50″ N, 73°18′52″ E~104°46′59″ E), involving Tibet, Qinghai, and some regions of Xinjiang, Gansu, Sichuan, and Yunnan in China (Figure 1). The larvae samples were identified by external morphology and molecular technology. Morphological characteristics, such as color of pronotum and markings on intersegmental membranes, could efficiently and quickly identify these two larval species [22]. Using the Biospin insect genomic DNA extraction kit (Bioer Technology Co., Ltd., Hangzhou, Chian), 13 last instar larvae from each sampling location were selected, and the DNA was extracted. Cytochrome c oxidase subunit I (*COI*: LCO1490: 5′-GGTCAACAAATCATAAAGATATTGG-3′ and HCO2198: 5′-TAAACTTCAGGGTGACCAAAAAATCA-3′) was amplified [43].

GPS (Garmin ETREX221x, Garmin, Shanghai, China) was used to record the information on the sampling points. With a combination of the Global Biodiversity Information Facility (GBIF, https://www.gbif.org, accessed on 18 November 2023) with the literature records [22,25,26,27,30,33,35,36,37,44,45,46], the distribution information of species (including geographical coordinates and altitude) was analyzed by *t*-test using the SPSS v27.0 package. In order to avoid the overfitting and spatial autocorrelation issues caused by clustered distribution data, ENMTools v1.1.0 (http://enmtools.blogspot.com, accessed on 21 January 2024) was used to filter distribution data, with 38 distribution points of *Gm* [47] and 45 distribution points of *Gq* individually obtained (Table S1 and Figure 1). The elevation data and map information used in this study were sourced from the Geographic Spatial Data Cloud (http://www.gscloud.cn, accessed on 20 January 2024) and the National Basic Geographic Information Database (https://data.tpdc.ac.cn/home, accessed on 20 January 2024), respectively. Meanwhile, the boundary range data and land use data of the Qinghai-Tibet Plateau came from the National Tibetan Plateau Data Center (https://data.tpdc.ac.cn/home, accessed on 22 January 2024) and the website (https://zenodo.org, accessed on 23 January 2024), respectively [48,49].

### 2.2. Population Density Survey

In this study, population surveys of grassland caterpillars were conducted at their most active times of each day between 10:00 and 16:00. Surveys were conducted five times repeatedly, with each time conducted every ten days [50,51,52]. Three 10 m × 10 m quadrat frames were randomly placed in each survey plot. In order to mitigate the influences of observation errors within a single time period on the research outcomes, this study statistically analyzed the average value of results of population surveys conducted five times using SPSS with reference to the relevant methods introduced by Song et al. [52] and Pan et al. [53].

### 2.3. Selecting and Screening Environmental Variables

A total of nineteen bioclimatic variables and one elevation variable with a resolution of 2.5 min during seven periods (Last Inter-Glacial (130,000 to 115,000 years ago), Last Glacial Maximum (22,000 years ago), Mid-Holocene (6000 years ago), current period, and future period (2041–2060, 2061–2080, 2081–2100)) were obtained from the WorldClim database (https://www.worldclim.org, accessed on 8 December 2023). Meanwhile, ArcGIS v10.4 software was used to exact two aspect and slope variables from the elevation variable, with a total of twenty-two environmental variables obtained (Table S2). A CCSM4 atmospheric circulation model was selected for the simulation of variables during the LIG, LGM, and MH periods. The Shared Socioeconomic Pathway (SSP) proposed in the Sixth Coupled Model Intercomparison Project (CMIP6) was selected for the simulation of future climate variables. The BCC-CSM2-MR atmospheric circulation model developed by the Beijing Climate Center was also used in the simulation [54]. Three scenarios, namely, the low-pressure scenario (SSP126), the medium-pressure scenario (SSP370), and the high-pressure scenario (SSP585), were applied in this study. SSP126, SSP370, and SSP585 refer to different Shared Socioeconomic Pathways (SSPs), which represent greenhouse gas emission scenarios under varying socioeconomic conditions.

Introducing an excessive number of environmental variables into the prediction of an ecological niche model could result in overfitting issues. With a too-high complexity, the model tends to overly adapt to the specific characteristics of training data, thus leading to its reduced generalization capacity when processing new data. When the model involves too many variables, it cannot grasp the fundamental patterns that can be broadly applied to new datasets [39,55]. In this study, initial modeling was conducted using the BIOMOD2 model to obtain the contribution rates of all climate variables. Subsequently, SPSS v27 was employed to conduct a Pearson correlation analysis on all environmental variables (Figure S1). After the removal of environmental variables with |r| > 0.8 and low contribution rates [56], eight primary environmental variables for each species were finally selected for subsequent model analyses (Table S2). Response curves reflect the quantitative relationships between the probability value of occurrence and the suitable ranges of environmental variables. A probability value of occurrence higher than 0.5 indicates favorable ranges of environmental variables for species survival [39].

### 2.4. BIOMOD2 Model Construction

The BIOMOD2 model provides an integrated model calculation capability for analyzing and interpreting model outputs more comprehensively, which makes this integration model exhibit better prediction performance than single models [57,58]. Based on those environmental variables and distribution data selected, modeling analyses using the BIOMOD2 package v3.5.1 (https://cran.r-project.org/src/contrib/Archive/biomod2/, accessed on 21 December 2023) in the R v4.2.3 (https://cran.r-project.org/bin/windows/base/old/4.2.3/, accessed on 21 December 2023) language were individually performed on the species *Gm* and *Gq*. Due to the failed operation of the GAM model, subsequent analyses were conducted using the remaining nine models (ANN, CTA, FDA, GBM, GLM, MARS, MAXENT, RF, and SRE). Species presence points and pseudo-absence points are required for the construction of the BIOMOD2 model. In order to better simulate actual species distribution and reduce spatial biases, a total of 1000 pseudo-absence points were randomly generated [47]. All nine models employed the default setting of the BIOMOD2 model, with 30% of data designated for the test dataset and the remaining 70% of data designated for the training dataset. Each model was operated four times repeatedly. A weighted average method was used to assign different weights to single models according to their performances, with a weighted average of prediction values of all single models calculated as the final prediction value.

$$\Sigma \left(\mathrm{Wi}\times \mathrm{Pi}\right)∕\Sigma Wi$$

Wi represents the weight assigned to each model, and Pi represents the prediction value of each single model.

Typically, each single model is assigned a weight based on its evaluation metrics, such as AUC, TSS, and KAPPA. In general, single models with better performances are assigned higher weights. The weighted average method can better leverage the comparative advantages of different models and improve the prediction accuracy of integration models [59,60]. In this study, the prediction results of nine models were integrated using the weighted average method, with a final integrated result achieved.

### 2.5. Model Accuracy Assessment

AUC is an index (with a range from 0 to 1) for assessing the classification capacities of models. It reflects the area under the ROC curve, and an AUC value close to 1 indicates a better classification capacity of the model, that is, the model can more precisely distinguish the presence locations of species from those locations with no species presence [61]. An AUC value of no more than 0.5 indicates that the prediction performance of the model is inferior to that of a random prediction, while an AUC value higher than 0.5 indicates the certain prediction superiority of the model.

$$\mathrm{AUC}=\int \mathrm{TPR}\left(\mathrm{FPR}\right)\;\mathrm{dFPR}$$

TPR represents a true positive rate, and FPR represents a false positive rate.

The KAPPA coefficient is an index measuring the classification accuracy of models with the influences of random consistency taken into account. The values of KAPPA range from −1 to 1. A KAPPA value of 1 indicates complete consistency, and a KAPPA value of 0 indicates a consistency that is randomly caused, with negative values of KAPPA indicating consistency that is less remarkable than that of random expectation. In the evaluation of an ecological niche model, the KAPPA coefficient can be used to measure the consistency between predicted distribution and actual distribution [62].

$$\mathrm{Kappa}=\left(Po-Pe\right)∕\left(1-\mathrm{Pe}\right)$$

Po represents the observed proportion of consistency, and Pe represents the anticipated proportion of random consistency.

TSS is a composite index involving the sensitivity and specificity of models. The values of TSS range from −1 to 1. A TSS value of 1 indicates a perfect prediction, and a TSS value of 0 indicates a prediction result that is inferior to that of random prediction, with negative TSS values indicating poor prediction performances of models. TSS is especially suitable for the scenarios of unbalanced datasets (with unequal quantities of samples with and without species presence) [63].

TSS=TPR+TNR−1

TPR represents a true positive rate, and TNR represents a true negative rate.

Independent test datasets were used in this study to calculate all the above indices to ensure the objectivity and accuracy of evaluation results. A comprehensive use of AUC, KAPPA, and TSS indices can thoroughly assess the prediction performances of ecological niche models, thus verifying the reliability and effectiveness of these models in real applications.

### 2.6. Correlation Analysis between Current Potential Distribution Area Prediction and Land Use Types

After the modeling of two species using the BIOMOD2 integration model was conducted, the integrated results were visualized using the ArcGIS v10.4 software and subsequently reclassified using a natural breaks classification method [64]. The potential distribution areas of those two sibling species in the QTP region were divided into the low suitability, medium suitability, and high suitability habitats and the no suitability area. Floating-point fields were added to the file attribute tables for all reclassified prediction results. The areas of all suitable habitats were then calculated using the pixel sizes and the VB script in the field calculator. The areas of suitable habitats during the paleoclimate and future periods were also calculated using the VB script. The intersection zones of potential habitats of those two grassland caterpillars and their land use types were obtained using the overlay module in ArcGIS, and the areas of these zones were calculated using the VB script. A Pearson correlation analysis and linear fitting of areas of different land use types and potential habitats were performed using the Origin software v2019b 32Bit to achieve data visualization. In the fitting,

$$R^{2}=1-\left(\mathrm{SSres}∕\mathrm{SStot}\right)$$

SSres represents the sum of squares of residuals, and SStot represents the total sum of squares.

### 2.7. Shifting Analysis of Distribution Centers during Different Time Periods

Based on the files of reclassified binary prediction results, this study calculated the distribution centers of those two species in question using the Distribution Changes Between Binary SDMs module in the SDMtoolbox (http://www.sdmtoolbox.org, accessed on 23 December 2023), thus achieving data visualization using ArcGIS [65].

## 3. Results

### 3.1. Comparison of Geographic Distribution and Population Density of Two Grassland Caterpillars

Survey results of species geographic distribution show that *Gm* is predominantly distributed in the alpine meadows of the northeastern boundary area of the QTP region (Figure 1). This species primarily feeds on such plants as *Elymus nutans* (Griseb), *Stipa aliena* (Keng), and *Kobresia humilis* (C.A.Mey. ex Trautv.). The average elevation of its distribution areas is approximately 3100 m. The *Gq* is predominantly distributed in the alpine meadows of the central and eastern areas of the QTP region (Figure 1). This species primarily feeds on such plants as *E. nutans*, *K. humilis*, and *K. capillifolia* (Decne.). The average elevation of its distribution areas is approximately 4000 m. The distribution areas of these two species exhibit extremely significant differences in elevations (t = −11.04; *p* < 0.001), as well as latitudes and longitudes (t = 14.29; *p* < 0.001, t = 7.34; *p* < 0.001) (Table S1 and Figure S2). Their population density and species accumulation curves show that the species richness of *Gq* is higher than that of *Gm*. Comprehensive results show that these two species present differences in their geographic distribution, distribution elevations, and population density (Table S1 and Figure S2).

### 3.2. Accuracy Evaluation of Single Models and Integration Models

**Figure 2:**
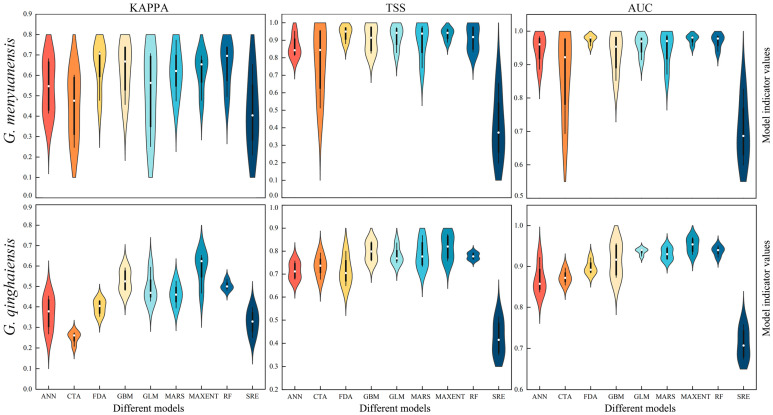
Evaluation indicators of KAPPA, TSS, and AUC for different models. evaluation indicators for different models.

This study compared the KAPPA, TSS, and AUC values of nine single models and the BIOMOD2 integration models for two species (Table S3 and Figure 2). The results show that among all nine single models of the Gm, the RF, FDA, and MAXENT models exhibit the highest average values of KAPPA, TSS, and AUC. In contrast, the CTA and SRE models showed greater variability in their indices. The integration models outperformed single models across all metrics, achieving excellent levels for TSS and AUC and a good level for KAPPA (Table S3 and Figure 2). For Gq, the MAXENT model had the highest average values for KAPPA, TSS, and AUC, while the CTA model had the lowest KAPPA value and the SRE model had the lowest TSS and AUC values. The ANN, FDA, and GBM models exhibited varying degrees of scattered KAPPA, TSS, and AUC values (Table S3 and Figure 2). Overall, the integration models demonstrated higher accuracy and stability in species prediction compared to single models.

### 3.3. Influence Analysis of Dominant Environmental Factors

**Figure 3:**
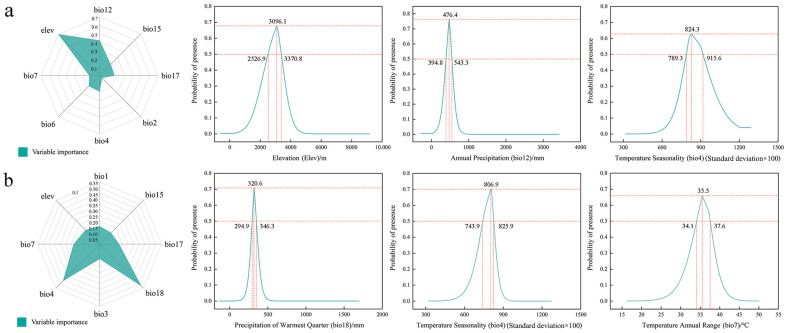
Importance and response curve of environmental factors: (**a**) *Gm* and (**b**) *Gq.*

According to the results of contribution rates and permutation importance of environmental variables, the dominant environmental factors affecting the distribution of the *Gm* are elevation (elev), annual precipitation (bio12), and temperature seasonality (bio4) (Table S2 and Figure 3a). An elevation range of 2526.9–3370.8 m is favorable for the survival of the *Gm*, with an optimal elevation value of 3096.1 m. Meanwhile, an annual precipitation range of 394.8–543.3 mm is favorable for the survival of the species, with an optimal annual precipitation of 476.4 mm. A temperature seasonality range of 789.3–915.6 is favorable for the survival of the species, with an optimal value of 824.3 (Figure 3a). The dominant environmental factors affecting the distribution of the *Gq* include precipitation of the warmest quarter (bio18), temperature seasonality (bio4), and temperature annual range (bio7), with importance values of 0.51863, 0.457648, and 0.228352, respectively (Table S2 and Figure 3b). A precipitation range of 294.9–346.3 mm during the warmest quarter is favorable for the survival of this species, with an optimal precipitation amount of 320.6 mm. Meanwhile, a temperature seasonality range of 743.9–825.9 is favorable for the survival of the species, with an optimal value of 806.9. A temperature annual range of 34.1 °C–37.6 °C is favorable for the survival of the species, with an optimal value of 35.5 °C (Figure 3b). Comprehensive results show that the distribution of these two species is primarily affected by the temperature and precipitation factors, with the topographical factor being a secondary factor, and that the distribution of the *Gm* is also affected by the elevation factor (Table S2). The results also verify the hypothesis that the spatial distribution patterns of these two sibling species with similar morphological characteristics, host preferences, and life history characteristics could be affected by the same ecological factors.

### 3.4. Prediction Results of Integration Models on the Current Potential Distribution Areas of Two Grassland Caterpillars

**Figure 4:**
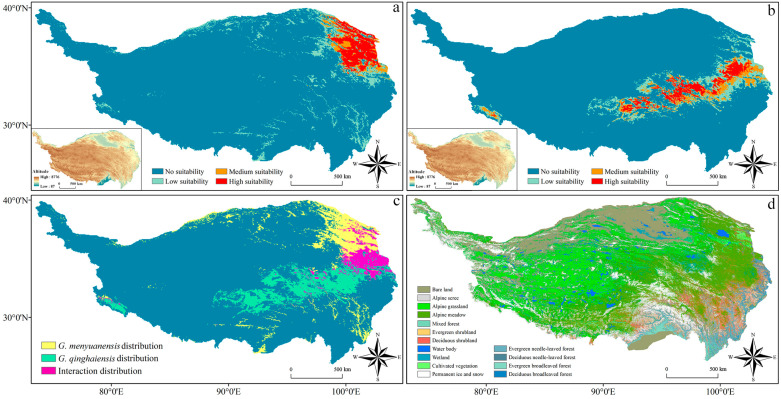
Current period prediction results under integrated model: (**a**) prediction results of *Gm*, (**b**) prediction results of *Gq*, (**c**) intersection situation of suitable habitats, and (**d**) land cover map of the Qinghai-Tibet Plateau.

The prediction results of integration models on the current potential habitats of these two grassland caterpillars on the QTP are shown in Figure 4. It can be seen that the potential habitats of the *Gm* are primarily concentrated in the northeastern boundary area of the QTP region. Specifically, these habitats are located in the northeastern part of Qinghai and some regions in Gansu. In addition, the medium and high-suitable habitats of this species are concentrated in Qinghai, with an area of 117,300 km^2^ (Table 1 and Figure 4a). The unsuitable zones of this species have a total area of 2.2513 million square meters, accounting for 88.79% of the total QTP area. The potential habitats of the *Gq* are primarily concentrated in the central and eastern areas of the QTP region. Specifically, these habitats are located in southern Qinghai and eastern Tibet, including Zanda County and Pulan County. With a total area of 139,000 km^2^, the medium and high-suitable habitats of this species are concentrated in southern Qinghai and eastern Tibet, accounting for 5.48% of the total QTP area (Table 1 and Figure 4b). In addition, the unsuitable zones of this species cover a total area of 2.2456 million square meters, accounting for 88.56% of the total QTP area. The potential distribution overlapping zones of these two species are concentrated in Zeku County, Tongde County, Maqin County, and Henan County of Qinghai and parts of Xiahe County in Gansu, with a total area of 78,070 km^2^ (Figure 4c). Land use results show that the potential habitats of these two species are primarily concentrated in the alpine meadow areas on the QTP (Figure 4d). Comprehensive results indicate that the suitable habitats of these two species are primarily concentrated in the alpine meadow areas of the QTP, with relatively small proportions of their areas in the total QTP area. Compared to the potential habitats of the *Gq*, those habitats of the *Gm* are inclined to be located in the low-elevation northeastern boundary area of the QTP, with a small proportion of their total area in the QTP area.

### 3.5. Correlations between Current Different Land Use Types and Prediction Results of Integration Models on Distribution of Species Potential Habitats

**Figure 5:**
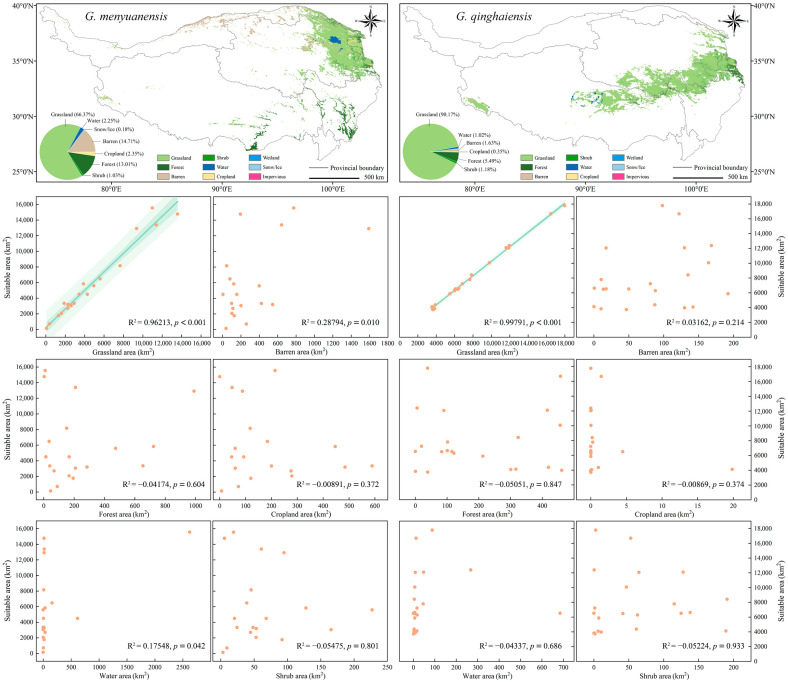
Land use situation of *Gm* and *Gq* suitable habitat areas and Pearson correlation analysis and linear fitting between suitable habitat areas and different land use types.

Correlation analysis results of land use types of distribution zones of two grassland caterpillars with their potential habitat distribution are shown in Figure 5. It can be seen that among all land use types, followed by the areas of barren, forest, cropland, water, shrubs, ice and snow, wetland, and impervious, the areas of grassland in the distribution zones of the *Gm* and *Gq* present the highest proportions of the total QTP area, accounting for 66.37% and 90.17% of that area, respectively (Figure 5). Correlation analysis results of areas of different land use types with the areas of potential habitats of species indicate that there are positive correlations between the areas of suitable habitats of those two species and their grassland areas (R^2^ = 0.96213, *p* < 0.001; R^2^ = 0.99791, *p* < 0.001), with no correlations between the areas of their suitable habitats and the areas of other land use types (Figure 5). These results further verify the hypothesis that the spatial distribution patterns of these two sibling species with similar morphological characteristics, host preferences, and life history characteristics could be affected by the same land use types.

### 3.6. Prediction Results of Integration Models on Species Potential Habitats during the Paleoclimate Period

**Figure 6:**
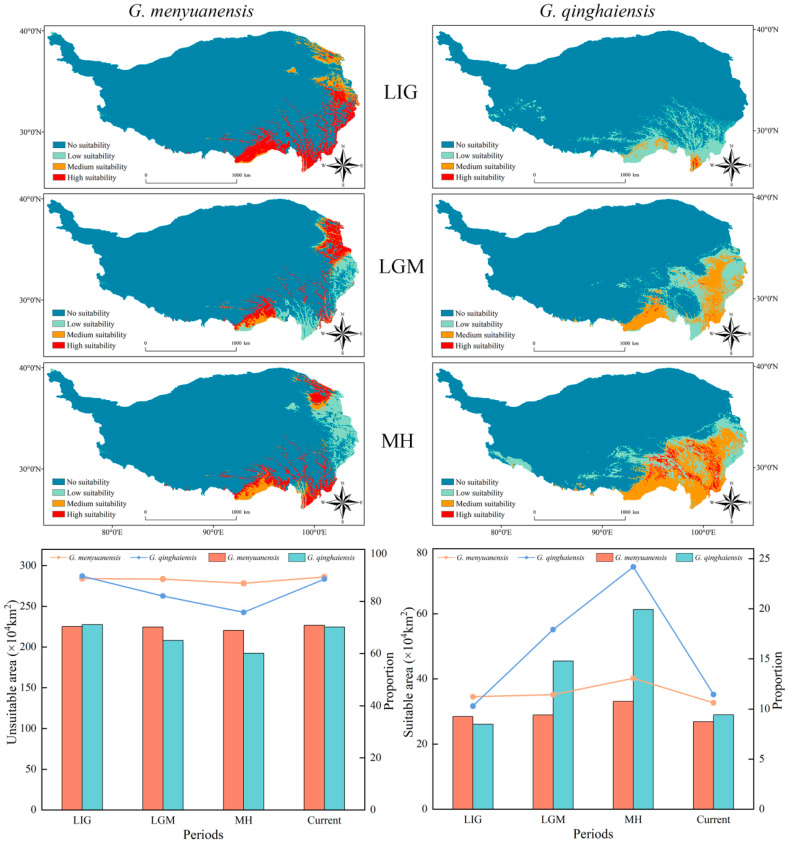
Predicted results of *Gm* and *Gq* during the paleoclimate period. LIG: predicted results for the Last Interglacial period. LGM: predicted results for the Last Glacial Maximum period. MH: predicted results for the Mid-Holocene. Bar chart: area of suitable and unsuitable habitats for *Gm* and *Gq*. Line chart: proportion of suitable and unsuitable area for *Gm* and *Gq*.

The prediction results of integration models on the species potential habitats during the paleoclimate period (LIG, LGM, and MH) are shown in Figure 6. It can be seen that, during the LIG period, the potential habitats of the *Gm* were concentrated in the eastern and southeastern boundary areas of the QTP. Specifically, its high-suitable habitats were concentrated in the southeastern boundary area of the QTP, with a total area of 188,100 km^2^. Its medium and low-suitable habitats were concentrated in the northeastern boundary area of the QTP, with a total area of 96,200 km^2^, making all of its suitable habitats account for 11.21% of the total QTP area (Table 1 and Figure 6). The potential habitats of the *Gq* during the same period were concentrated in the southeastern boundary area of the QTP, with a small total area of its high-suitable habitats, which is only 2500 km^2^. The medium-suitable habitats of this species were concentrated in the southeastern part of the QTP. Its low-suitable habitats were located in the southeastern area of the QTP, with a small portion being located in the western part of the QTP. The total area of its suitable habitats was 260,700 km^2^, accounting for 10.28% of the total area of the QTP. This area was slightly lower than the total area of suitable habitats of the *Gm* (Table 1 and Figure 6).

During the LGM period, the locations of potential habitats of the species *Gm* were similar to those of the species during the LIG period. Some parts of its high-suitable habitats were transferred to the northeastern boundary area of the QTP, with a slight decrease in the total area of its high-suitable habitats (which is 116,600 km^2^) compared to that area during the LIG period. The total area of its medium and low-suitable habitats (which is 172,900 km^2^) was larger than that area during the LIG period. During the LGM period, the total area of this species’ suitable habitats was 289,500 km^2^, accounting for 11.42% of the total area of the QTP. During the same period, the total area of potential habitats of the *Gq* was significantly larger than that area during the LIG period. Its low-suitable habitats were mostly transferred to the southeastern boundary area of the QTP, with only a small portion still remaining in the western part of the region. The total area of suitable habitats for this species was 454,400 km^2^, accounting for 17.92% of the total area of the QTP (Table 1 and Figure 6).

The locations of potential habitats of the *Gm* during the MH period were similar to those of the species during the LGM period. In the northeastern boundary area of the QTP, the high-suitable habitats of this species shrank, and its low-suitable habitats expanded. Meanwhile, its high-suitable habitats in the southeastern boundary area of the QTP expanded, with the total area of its high-suitable habitats reaching 122,300 km^2^. During the MH period, the total area of suitable habitats for this species expanded to 330,800 km^2^, accounting for 13.04% of the total area of the QTP (Table 1 and Figure 6). During the same period, the potential habitats of the *Gq* expanded significantly compared to those habitats of the species during the LGM period. The total area of its high-suitable habitats increased to 61,000 km^2^, with its expanding low-suitable habitats in the southwestern boundary area of the QTP. During the MH period, the total area of suitable habitats for this species increased to 612,900 km^2^, accounting for 24.17% of the total area of the QTP (Table 1 and Figure 6).

Comprehensive results show that during the paleoclimate period, the potential habitats of the *Gm* were concentrated in the eastern and southeastern boundary areas of the QTP (northern Hengduan Mountains). During that period, the locations of suitable habitats for this species were significantly different from those of the species during the current period. Meanwhile, the total area of its suitable habitats during the paleoclimate period is lower than the area of the species during the current period. Similarly, the potential habitats of the *Gq* were concentrated in the southeastern boundary area of the QTP (northern Hengduan Mountains). Compared with its potential habitats during the current period, those habitats of this species during the paleoclimate period presented a trend of expansion. After reaching its peak level during the MH period, the total area of suitable habitats for this species continuously decreased. Overall, during the glacial period, the areas of its suitable habitats increased with rising temperatures, presenting a pattern of LIG < LGM < MH. Areas of suitable habitats of the *Gq* and *Gm* during the LGM period are significantly different from those during the MH period (*Gq* > *Gm*) (Table 1 and Figure 6). These results verify the hypothesis that grassland caterpillar species in high-altitude regions are more susceptible to temperature variations in their suitable habitat distribution than those species in low-altitude regions and that the Hengduan Mountains provided a refuge for multiple species during the glacial period.

### 3.7. Prediction Results of Integration Models under Different Climate Scenarios during the Future Period

**Figure 7:**
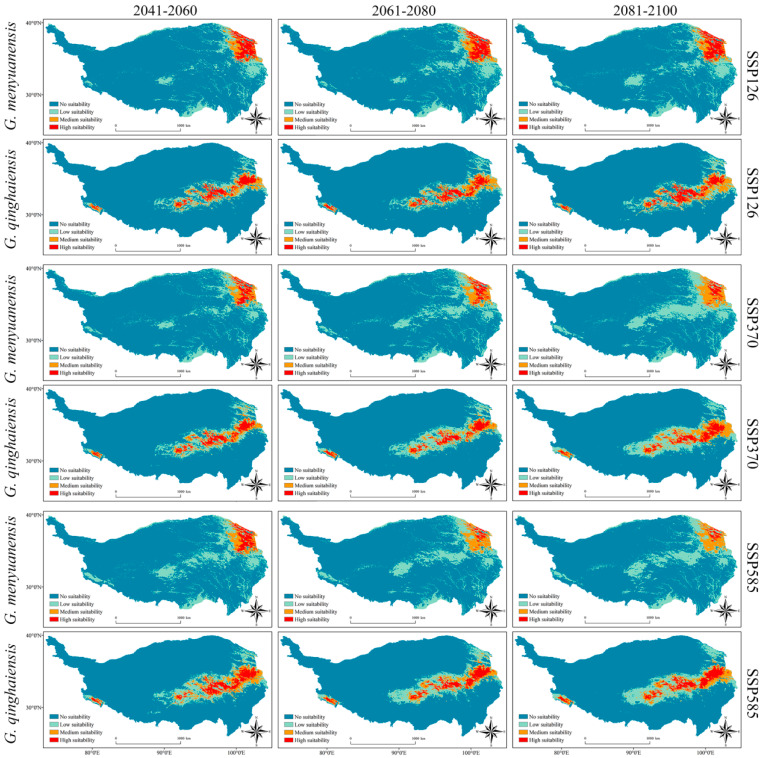
Predicted results of *Gm* and *Gq* during the future period. 2041–2060, 2061–2080, and 2081–2100: different periods in the future. SSP126: The low-pressure scenario. SSP370: the medium-pressure scenario. SSP585: the high-pressure scenario.

The prediction results of species’ potential habitats during the future period (2041–2060, 2061–2080, and 2081–2100; SSP126, SSP370, and SSP585) are illustrated as follows. Under all three climate scenarios, the potential habitats of both species will present roughly similar distribution patterns to those during the current period. Specifically, the high-suitable habitats of the *Gm* and *Gq* will be concentrated in the northeastern boundary area and the central and eastern parts of the QTP, respectively (Figure 7).

Under the climate scenario of SSP126, compared with those areas during the present period, the areas of high-suitable habitats of the *Gm* will gradually decrease, with gradually increased areas of medium and low-suitable habitats of the same species. During those three time periods (2041–2060, 2061–2080, and 2081–2100), the total areas of suitable habitats of the species will reach 383,200 km^2^, 451,000 km^2^, and 464,500 km^2^, accounting for 15.11%, 17.78%, and 18.32% of the total QTP area, respectively (Table 1 and Figure 7). With the total area of its suitable habitats lower than that of the *Gm*, areas of the high, medium, and low-suitable habitats of the *Gq* will all gradually increase, accounting for 13.58%, 13.90%, and 15.38% of the total QTP area, respectively (Table 1 and Figure 7).

Under the climate scenario of SSP370, compared with those areas during the present period and under the scenario of SSP126, the areas of high-suitable habitats of the *Gm* will gradually decrease. The total areas of its suitable habitats during all three time sections will slightly increase and be larger than those areas during the present period and under the scenario of SSP126, accounting for 15.70%, 18.30%, and 22.42% of the total QTP area, respectively (Table 1 and Figure 7). Areas of suitable habitats for the *Gq* will increase with varying degrees compared with those areas during the present period. Compared to those areas under the scenario of SSP126, only areas of high-suitable habitats and some medium-suitable habitats of this species will decrease. During those three time sections, all total areas of its suitable habitats will continue to increase, accounting for 14.15%, 15.34%, and 18.37% of the total QTP area, respectively (Table 1 and Figure 7).

Under the climate scenario of SSP585, except the areas of high-suitable habitats of the *Gm* during the periods of 2041–2060 will be slightly larger than those areas under the scenario of SSP370, during other time sections, these total areas will be all smaller than those areas during the present period and under the scenarios of SSP126 and SSP370. Meanwhile, areas of medium and low-suitable habitats for this species will present an increasing trend. During all three time sections, the total areas of suitable habitats for this species will present a decreasing trend. Except that the total areas of its suitable habitats during the time section of 2081–2100 will be smaller than those areas under the scenario of SSP370, during other time sections, these total areas will be all greater than those areas during the present period and under the scenarios of SSP126 and SSP370. In addition, the total areas of suitable habitats for this species during those three time sections will account for 20.13%, 18.83%, and 19.76% of the total QTP area, respectively (Table 1 and Figure 7). Under the scenario of SSP585, the total areas of suitable habitats of the *Gq* will be greater than those areas during the present period and under the scenarios of SSP126 and SSP370, with its total areas of suitable habitats during those three time sections reaching 446,800 km^2^, 431,100 km^2^, and 471,200 km^2^, respectively (Table 1 and Figure S3).

Comprehensive results show that potential habitats for both species during the future period will present a trend of increase. Under three different climate scenarios and during different time sections, potential habitats of the species *Gm* will be all larger than those of the *Gq*. Meanwhile, the potential distribution of these two sibling species will increase with intensifying pressure (Table 1 and Figure S3).

### 3.8. Changes in Species Distribution Centers during Different Periods

**Figure 8:**
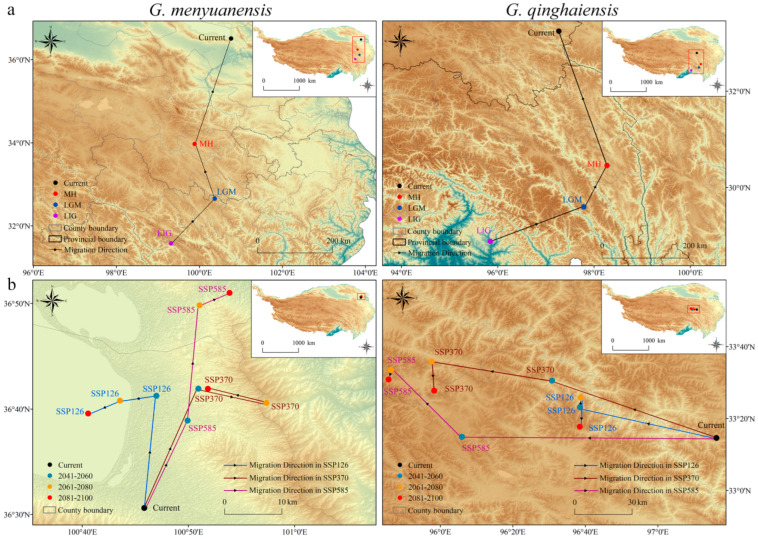
Migration patterns of distribution centers for *Gm* and *Gq* during different periods. (**a**) Migration patterns of distribution centers for *Gm* and *Gq* during the paleoclimate period. LIG: Last Interglacial period. LGM: Last Glacial Maximum period. MH: Mid-Holocene. (**b**) Migration patterns of distribution centers for *Gm* and *Gq* during the future period. 2041–2060, 2061–2080, and 2081–2100: different periods in the future. SSP126: the low-pressure scenario. SSP370: the medium-pressure scenario. SSP585: the high-pressure scenario.

Shifting analysis results of species distribution centers during the paleoclimate and future periods are illustrated as follows. During the paleoclimate period, the distribution center of the *Gm* was located in the central and eastern parts of the QTP (Figure 8a). Specifically, during the LIG, LGM, and MH periods, distribution centers of this species were located in areas of current Baiyu County and Seda County of Sichuan, and current Gande County of Qinghai, respectively. Subsequently, its distribution center moved northward continuously, finally reaching the area of current Gonghe County in Qinghai (100.76° E, 36.51° N) (Figure 8a). Among all these periods, the distribution center of this species presented the longest shifting distance of 293.76 km during the MH period. The distribution center of the *Gq* during the paleoclimate period was located in the southern part of the QTP. Specifically, during the LIG, LGM, and MH periods, its distribution centers were located in the areas of current Motuo County, Zuogong County, and Chaya County of the Tibet Autonomous Region, respectively (Figure 8a). Subsequently, the distribution center of this species continuously moved northward, finally reaching the area of current Chengduo County in Qinghai (97.27° E and 33.24° N). Among all these periods, its distribution center presented the longest shifting distance of 324.18 km during the MH period (Table S4 and Figure 8a).

During the future period, the distribution center of the *Gm* will be located in the northeastern boundary area of the QTP (specifically, Gonghe County, Huangyuan County, and Haiyan County) (Figure 8b). Under the SSP126 scenario, its distribution center will shift westward as a whole and be located in Gonghe County. Under the SSP370 scenario, the distribution center of this species will move eastward as a whole. Except that its distribution center will move into Huangyuan County (100.96° E, 36.68° N) during the time section of 2061–2080, it will remain in Gonghe County during other time sections (Figure 8b). Under the SSP585 scenario, the distribution center of the *Gm* will shift northward as a whole, presenting the longest distance of shifting. During the time section of 2061–2100, its distribution center will move into Haiyan County with a shifting distance of 41.56 km (Table S4 and Figure 8b). During the future period, the distribution center of the *Gq* will be located in the central part of the QTP (Zhiduo County and Yushu City) (Figure 8b). Under all three climate scenarios, its distribution center will shift westward. Except that the distribution center will be located in Zhiduo County during the time section of 2061–2100 under the SSP370 and SSP585 scenarios, it will be located in Yushu City under all other conditions. Under the SSP585 scenario, the distribution center of this species will present the longest shifting distance of 159.89 km (Table S4 and Figure 8b).

Comprehensive results show that during the paleoclimate period, the distribution centers of both species shifted northward (from the low-latitude northern part of the Hengduan Mountains to the high-latitude northeastern part of the Qinghai-Tibet Plateau), with the longest shifting distances during the MH period. In the future period, under those three different climate scenarios, the distribution centers of the *Gm* will shift northward first and then move towards the west, east, and north, respectively. Under all three climate scenarios, the distribution center of the *Gq* will consistently shift westward (Figure 8b). The migration ranges of both species will be relatively small, with their longest migration distances occurring under the SSP585 scenario (Table S4 and Figure S4).

## 4. Discussion

By combining the geographic distribution information of the *Gm* and *Gq* on the Qinghai-Tibet Plateau with 19 bioclimatic variables, 3 topographical variables, and land use data, this study predicted, compared, and analyzed the potential habitats of these two species using the BIOMOD2 integration model. In addition, the superiority of multiple assessment indices (KAPPA, TSS, and AUC) in the integration model was clarified (Table S3 and Figure 2). Single models such as Random Forest (RF), Functional Discriminant Analysis (FDA), and Maximum Entropy Model (MaxEnt) perform well on specific metrics. However, integration models can effectively integrate the advantages of different models and reduce the uncertainty and biases of prediction results (Table S3 and Figure 2). This is consistent with the study results of Lissovsky et al. [66]. The stability and accuracy demonstrated by integration models are of particular importance in ecological and biogeographical applications. Data in these fields are highly complex and variable [41]. This study revealed that the discreteness of single models in prediction performance could impact the generalization capacities and practical application value of these models [67]. Integration models demonstrate superior performance in predicting the distribution of grassland caterpillars compared with single models. These models can not only improve the prediction accuracy and stability of pest distribution zones but also provide robust scientific support for designating and managing pest prevention and control areas. Future studies can further explore how to optimize model structures and parameters for better adaptation to complex ecological environments and apply these models to different ecosystems and more species.

Climate and topographical factors are key factors in determining the distribution of species [68,69]. Unique high-altitude climate conditions, such as low temperatures and sparse oxygen, could restrict the distribution of many species. However, species adapting to these conditions can find suitable habitats in such areas [27,70] Temperature and precipitation have significant impacts on species distribution and community composition [71,72]. Closely related species share many genetic genes, which lead to their identical responses to various environmental pressures during their evolution processes. Therefore, these species could demonstrate consistent environmental adaptability and ecological requirements [73]. The results of this study show that there are differences in geographic distribution elevations and species richness between two closely related species (*Gq* > *Gm*; Table S1 and Figure 1). However, the distribution of their suitable habitats is primarily affected by significant environmental factors such as annual precipitation and temperature seasonality, with topographical factors serving as secondary factors (Table S2 and Figure 3 and Figure 4). This could be related to the identical ecological niches and physiological characteristics of both species [74]. The morphological characteristics, life history, and ecological adaptability of these two closely related species demonstrate a high level of adaptation to alpine environments. Their larvae primarily feed on alpine forage grasses with large consumption amounts, which poses significant challenges to prevention and control and severely threatens the stability of the grassland ecosystem in the QTP region [22,29,35]. With global warming, rising temperatures and increasing precipitation amounts have directly contributed to the increased host plant diversity in this region, thus leading to increased population density and expanded distribution areas of these two pests. The analysis results of dominant environmental factors in this study have provided technical support for monitoring and early warning of grassland caterpillars and facilitated the establishment of effective pest prevention and control areas.

Protecting grassland ecosystems is of crucial importance for maintaining biodiversity, playing a key role in controlling grassland pests, including caterpillars [75]. Prediction results of species’ potential habitats during the current period show that the *Gm* prefers to live in the northeastern low-altitude boundary area of the QTP region, while the *Gq* is more adaptable to the central and eastern high-altitude areas in the region. These two species present different adaptability to elevation and microclimate conditions (Figure 4). However, the distribution patterns of their suitable habitats are closely related to the distribution of alpine meadows (Figure 5). Land use analysis indicates that grassland makes up the largest proportion of the potential distribution areas of both species, with a significantly positive correlation between the areas of their potential habitats and grasslands (Figure 5). This has verified the core role of grassland ecosystems in maintaining the population density of grassland caterpillars. These study results have thus further validated our hypothesis; that is, the spatial distribution patterns of two closely related pests with similar morphological characteristics, host preferences, and life history characteristics are affected by identical ecological factors and land use types. Future studies should further explore the ecological requirements and adaptability of these pests and their potential impacts on grassland ecosystems. For example, their feeding preferences on different plant varieties, as well as how these preferences affect their distribution patterns and population dynamics, can be investigated [76].

The Hengduan Mountains are one of the hot topics of biodiversity research [77,78,79]. Due to the uplift of the Qinghai-Tibet Plateau, with significant changes in climate, geology, and biodiversity, the terrain of these mountains and their southwestern neighboring areas became extremely complex, making these areas a shelter for most species during the glacial period [80,81]. The current and past distribution patterns of herbivorous insects are closely related to climate conditions (especially those of the Pleistocene glacial epoch) [82,83]. Climate fluctuations during the Pleistocene glacial epoch, especially the alternation of glacial and interglacial periods, exerted a profound influence on the geographical distribution of herbivorous insects, resulting in the formation of multiple shelter areas [84,85,86]. Analysis results of species distribution models (SDMs) in this study show that during the paleoclimate period, the potential habitats of those two caterpillars were located in the eastern and southeastern boundary areas of the QTP. Compared with their potential habitats during the current period, these habitats expanded into the southern low-latitude areas of the Hengduan Mountains with increased areas of their distribution zones (Figure 6). The consistent and stable microclimate of the Hengduan Mountains helped species survive under the violent climate fluctuations during the glacial period, allowing them to migrate to habitats with more favorable conditions [87]. Subsequently, with temperature rising and time progression, the distribution centers of these species moved towards high-latitude areas in the northeast, and their suitable habitats all expanded. In particular, suitable habitats for the *Gq* in low-latitude areas increased significantly during the MH period (Figure 6 and Figure 8). Species migration constitutes a major biological response to climate change [88]. With the warming of the climate and the melting of ice sheets, populations of these two grassland caterpillars began to re-expand into areas previously covered by ice sheets. Stable climates provided favorable growth and reproduction conditions for the insect populations and promoted their recovery and expansion [89]. Distribution variations in suitable habitats of these two species caused by climate change not only impacted their geographic distribution but also possibly affected their genetic diversity and speciation [90]. The significant shifting of distribution centers of both species during the paleoclimate period (Table S4 and Figure 8) could be their responses to the situations of low temperatures and ice-sheet expansion during the glacial period for seeking warmer habitats and escaping from extreme climate conditions [91]. This further reflects their different ecological adaptation strategies to ancient climate changes [92].

Distribution changes in the future suitable habitats of grassland caterpillars will have a profound impact on ecosystems. Particularly, geographic expansion could result in new ecological interactions, and the expansion of their potential habitats can make these species invade new ecosystems and disrupt the existing ecological balance there [93]. Prediction results reveal the changes in the distribution patterns of suitable habitats of the *Gm* and *Gq* under future climate conditions. Under three representative climate scenarios (SSP126, SSP370, and SSP585), potential habitats of both species will exhibit a trend of increase. This is especially true under the SSP585 scenario that involves higher gas emissions (Table 1 and Figure 7). Global climate change could be the major cause of this situation. With global warming, previously cold high-latitude and high-altitude areas will become warmer, providing new habitable environments for grassland caterpillars [94]. Climate change could also affect precipitation patterns and alter vegetation distribution and productivity, thus further impacting food sources and habitat conditions of insects [18]. Prediction results show that medium and low-suitable habitats of the *Gm* will gradually increase, indicating that this species could expand to a broader range of areas. In comparison, high, medium, and low-suitable habitats of the *Gq* will all gradually increase, demonstrating stronger adaptability of this species to future climate change (Figure 7 and Figure S3). During the future period, the distribution center of the *Gm* will be located in the northeastern boundary area of the QTP (Gonghe County, Huangyuan County, and Haiyan County), presenting an overall northward shifting trend compared to its current pattern. Meanwhile, the distribution center of the *Gq* will be located in the central part of the QTP (Zhiduo County and Yushu City), presenting an overall westward shifting trend compared with its current pattern (Figure 8). These shifting trends could reflect the responses of both species to climate warming and humidity variations in the future to seek more favorable living conditions [94]. In this study, prediction results of integration models on species’ potential habitats can basically match the actual distribution ranges of those two grassland caterpillars on the QTP, thus further verifying the reliability and accuracy of these results. Therefore, this study has provided an important and valuable reference for designing prevention and control strategies for grassland caterpillars. Specifically, enormous control efforts should be applied in high-suitable habitats of grassland caterpillars, while major prevention efforts should be emphasized in low-suitable habitats of these grassland pests. However, model predictions in this research have not involved species adaptive capacities and biological interactions. It is hoped that this can be improved in future studies of ecological niche modeling [95,96].

## 5. Conclusions

Based on the BIOMOD2 integration model, this study comprehensively used actual species geographic distribution data, environmental factors, and land use data to compare and analyze the potential habitats of two closely related endemic species on the QTP and changes in their distribution centers during the paleoclimate, present, and future periods. The results show that the integration models of both species present significant advantages over their single models. The potential habitats of these two species are significantly affected by temperatures and precipitation. The areas of suitable habitats for both species are significantly and positively correlated with the area of grassland among all land use types in the QTP region. In addition, the distribution patterns of potential habitats of these two grassland caterpillars are significantly affected by the distribution of alpine meadows. Compared with those habitats during the current period, the potential habitats of both species during the paleoclimate period all expanded towards the Hengduan Mountains region with increased areas. Subsequently, with temperature rising and time progression, their distribution centers all shifted towards the northeastern high-latitude areas. Between these two species, the distribution of suitable habitats of the *Gq* was more susceptible to temperature changes during the glacial period. The following three hypotheses have been verified in this study: (1) The spatial distribution patterns of these two closely related species in question with similar morphological characteristics, host preferences, and life history characteristics could be affected by identical ecological factors and land use types. (2) Grassland caterpillars in high-altitude areas are more susceptible to temperature changes in the distribution of their suitable habitats than those species in low-altitude areas. (3) The Hengduan Mountains provided shelter for multiple species during the glacial period. Through precise identification of high-risk areas of grassland caterpillar damage, focus on targeted control measures for the high suitability habitats of the two grassland caterpillars. Therefore, it has provided an important reference for the conservation of alpine meadow ecosystems in the region.

## Figures and Tables

**Table 1:** Suitable habitat areas for different periods of *Gm* and *Gq.*

Species	Different Periods and Climate Scenarios	No Suitability (×10^4^ km^2^)	Low Suitability (×10^4^ km^2^)	Medium Suitability (×10^4^ km^2^)	High Suitability (×10^4^ km^2^)
*G. menyuanensis*	Current	226.68	15.14	3.69	8.04
	LIG	225.13	2.10	7.52	18.81
	LGM	224.60	12.48	4.81	11.66
	MH	220.47	14.74	6.12	12.23
	2041–2060SSP126	215.24	27.40	4.64	6.29
	2041–2060SSP370	213.74	30.73	5.27	3.81
	2041–2060SSP585	202.50	40.61	5.71	4.74
	2061–2080SSP126	208.46	33.44	5.08	6.59
	2061–2080SSP370	207.14	37.26	6.09	3.07
	2061–2080SSP585	205.80	39.08	7.10	1.58
	2081–2100SSP126	207.10	35.21	4.82	6.43
	2081–2100SSP370	196.70	46.51	7.95	2.41
	2081–2100SSP585	203.44	41.40	7.30	1.41
*G. qinghaiensis*	Current	224.56	15.10	7.32	6.58
	LIG	227.48	23.44	2.39	0.25
	LGM	208.12	24.72	20.35	0.37
	MH	192.27	24.25	30.95	6.10
	2041–2060SSP126	219.11	18.63	8.72	7.10
	2041–2060SSP370	217.68	20.37	8.60	6.92
	2041–2060SSP585	208.87	24.66	11.02	9.01
	2061–2080SSP126	218.31	18.81	9.53	6.91
	2061–2080SSP370	214.64	24.26	8.15	6.51
	2061–2080SSP585	210.45	25.52	9.52	8.07
	2081–2100SSP126	214.55	19.97	10.63	8.41
	2081–2100SSP370	206.96	26.91	11.83	7.86
	2081–2100SSP585	206.44	27.15	10.63	9.34

## Data Availability

The datasets supporting the conclusions of this article are included within the article.

## Supplementary Materials

The following supporting information can be downloaded at: https://www.mdpi.com/article/10.3390/insects15100781/s1, Table S1. Distribution point information. Table S2. Screening and importance of environmental variables. Table S3. Evaluation indicators for different models. Table S4. Centroid coordinates of distribution and migration distance. Figure S1. Correlation heatmap of environmental variables. Figure S2. Differences in longitude and latitude between two species. Figure S3. Suitable habitat areas for future periods of *Gm* and *Gq*. Figure S4. Distribution center migration distance.
