# Supplementary material for: Comparative Study of Potential Habitats for Two Endemic Grassland Caterpillars on the Qinghai-Tibet Plateau Based on BIOMOD2 and Land Use Data
Source: Insects. 2024 Oct 8;15(10):781. doi: 10.3390/insects15100781 (PMC11508900; doi:10.3390/insects15100781)
Supplement: Supplementary file 1 [file insects-15-00781-s001.zip › insects-3209212-supplementary.pdf]

Title: Comparative study of potential habitats for two endemic grassland caterpillars on the Qinghai-Tibet Plateau based on BIOMOD2 and land use data

Authors: Chuanji Li[1,2], Yunxiang Liu[1,2], Youpeng, Lai[1,2], Hainan Shao[1,*]

Supplementary Materials

Legends for Tables and Figures

Table S1 Distribution point information.

Species	Survey sites	Latitude	Longitude	Altitude(m)	Population density
G. menyuanensis	Datong County	37.3500	101.1833	3774	171
		36.9262	101.6949	2548	288
		37.2617	101.4163	2985	198
	Menyuan County	37.6167	101.2000	3112	171
		37.5000	101.3333	3027	243
	Haiyan County	37.0675	100.8677	3212	432
		37.1007	100.6727	3418	333
	Qilian County	37.8620	101.0702	3605	171
		37.9419	100.7447	3695	189
	Ledu District	36.3060	102.2393	2835	144
		36.6487	102.5631	3069	432
	Hualong County	36.1513	102.2014	3063	162
		36.0179	102.5724	2921	171
	Xunhua County	35.6113	102.6845	2810	369
		35.7796	102.2047	2842	315
	Tongren City	35.4719	101.9880	2724	333
		35.6478	101.8269	3278	279
	Guide County	36.0413	101.4059	2202	279
		35.8431	101.4010	2568	162
	Gonghe County	36.3598	100.7972	3180	369
		36.3558	99.4894	3170	450
		37.1457	99.4072	3264	108
	Xinghai County	35.8045	99.9009	3750	405
		35.2808	100.1769	3260	297

	Tongde County	35.0231	100.4647	3549	405
		34.8077	100.9479	3671	369
	Guinan County	35.4374	101.0078	3420	378
		35.8231	101.2406	3469	252
	Minle County	38.3977	100.6658	2592	396
		38.2468	100.7360	3383	135
	Sunan County	38.0333	101.3167	2973	288
		38.7966	99.5690	2453	378
		38.4115	100.4607	2558	126
	Tianjun County	37.6799	98.6884	3685	171
	Huzhu County	37.0085	102.1384	3127	207
	Jishishan County	35.7181	102.7596	2534	360
	Xiahe County	35.1388	102.4597	3008	387
G. qinghaiensis	Zaduo County	33.3512	94.5563	4548	189
		32.8344	95.3536	4162	324
	Yushu City	32.8900	96.7500	4246	372
		32.9500	96.5200	4655	396
		32.7564	96.4951	4324	552
	Baqing County	32.3115	93.8326	4439	420
	Chengduo County	33.3000	97.4500	4275	408
		33.6044	97.2493	4534	240
		33.3864	97.2208	4741	300
	Zhiduo County	34.2981	94.4275	4639	324
		33.7866	95.8191	4598	348
		34.0345	95.8088	4228	564
	Nangqian County	32.1897	96.4914	3646	312
		31.9002	95.9257	4075	552
	Maqu County	34.0978	100.8850	4423	516
		34.2394	101.1866	3478	228
		33.4298	102.3277	3442	288
	Maqin County	34.4409	100.2871	3740	480
		34.1474	99.2939	4323	564
	Gande County	33.9791	99.8444	4068	300
		34.1682	100.5337	3853	504
	Dari County	33.7361	99.7180	3985	144
		32.9465	99.3522	4508	420
	Zeku County	34.9916	101.4488	3759	156
		35.2402	100.9568	3430	300
	Henan County	34.8310	101.5354	3599	540
		34.3819	101.3227	3588	180

Luqu County	34.6129	102.4667	3171	144
	34.6137	102.9071	3151	240
Anduo County	32.2634	91.6961	4814	264
	31.9126	91.5119	4595	120
Nierong County	32.1243	92.3381	4631	564
	32.1528	92.8024	4572	480
Suo County	31.9166	93.7906	3994	576
	31.4576	94.5570	3836	408
Biru County	31.5005	93.7555	4177	480
	31.1942	94.1841	3962	408
Seni District	31.4623	92.0525	4518	240
	31.6305	92.2819	4521	588
Jiangda County	32.0065	97.5712	3757	216
Shiqu County	33.3196	97.6743	4291	420
	32.9594	98.0966	4204	396
	33.1663	98.1576	4009	348
Dingqing County	31.5933	95.0966	4149	324
	31.5221	95.2641	4247	372

Table S2 Screening and importance of environmental variables.

Environmental variable	Variable description	Unit	Variable importance	
			G. menyuanensis	*G. qinghaiensis*
bio1	Annual Mean Temperature	℃	\	**0.156611**
bio2	Mean Diurnal Range	℃	**0.037833**	\
bio3	Isothermality	\	\	**0.127333**
bio4	Temperature Seasonality	\	**0.188093**	**0.457648**
bio5	Max Temperature of Warmest Month	℃	\	\
bio6	Min Temperature of Coldest Month	℃	**0.178019**	\
bio7	Temperature Annual Range	℃	**0.120667**	**0.228352**
bio8	Mean Temperature of Wettest Quarter	℃	\	\
bio9	Mean Temperature of Driest Quarter	℃	\	\
bio10	Mean Temperature of Warmest Quarter	℃	\	\
bio11	Mean Temperature of Coldest Quarter	℃	\	\
bio12	Annual Precipitation	mm	**0.416315**	\
bio13	Precipitation of Wettest Month	mm	\	\
bio14	Precipitation of Driest Month	mm	\	\
bio15	Precipitation Seasonality	\	**0.183648**	**0.139315**
bio16	Precipitation of Wettest Quarter	mm	\	\
bio17	Precipitation of Driest Quarter	mm	**0.173741**	**0.170111**
bio18	Precipitation of Warmest Quarter	mm	\	**0.51863**
bio19	Precipitation of Coldest Quarter	mm	\	\
Elev	Elevation	m	**0.694037**	**0.174537**
Aspect	Aspect	°	\	\
Slope	Slope	°	\	\

Table S3 Evaluation indicators for different models.

Species	Model	KAPPA	TSS	AUC
G. menyuanensis	ANN	0.5475±0.1390	0.8628±0.0637	0.9478±0.0440
	CTA	0.4503±0.1706	0.7893±0.2113	0.8788±0.1341
	FDA	0.6518±0.1166	0.9380±0.0439	0.9755±0.0131
	GBM	0.6330±0.1351	0.9075±0.0799	0.9355±0.0613
	GLM	0.5210±0.2155	0.9210±0.0661	0.9583±0.0309
	MARS	0.6225±0.1221	0.8995±0.1084	0.9503±0.0540
	MAXENT	0.6203±0.0975	0.9340±0.0343	0.9758±0.0141
	RF	0.6563±0.1151	0.9115±0.0754	0.9703±0.0217
	SRE	0.4180±0.1871	0.3995±0.1945	0.6998±0.0970
	Ensemble model	0.9020	0.9420	0.9860
G. qinghaiensis	ANN	0.3693±0.0832	0.7143±0.0389	0.8685±0.0375
	CTA	0.2508±0.0299	0.7355±0.0471	0.8735±0.0173
	FDA	0.3983±0.0375	0.7145±0.0640	0.8973±0.0168
	GBM	0.5303±0.0553	0.7988±0.0439	0.9155±0.0431
	GLM	0.4898±0.0729	0.7805±0.0382	0.9368±0.0079
	MARS	0.46000±0.0542	0.7885±0.0624	0.9313±0.0156
	MAXENT	0.5920±0.0845	0.8175±0.0576	0.9520±0.0195
	RF	0.3693±0.0233	0.7780±0.0151	0.9383±0.0120
	SRE	0.2508±0.0624	0.4233±0.0779	0.7115±0.0394
	Ensemble model	0.8890	0.9240	0.9830

The value is the mean ± standard deviation.

Table S4. Centroid coordinates of distribution and migration distance.

Species	Period	Longitude	Latitude	Migration Distance (km）
G. menyuanensis	LIG	99.32	31.58	155.69
	LGM	100.37	32.65	152.81
	MH	99.89	33.97	293.76
	Current	100.76	36.51	0
	SSP126 (2041-2060)	100.78	36.69	19.81
	SSP126 (2061-2080)	100.73	36.68	5.15
	SSP126 (2081-2100)	100.68	36.66	5.00
	SSP370 (2041-2060)	100.85	36.70	22.32
	SSP370 (2061-2080)	100.96	36.68	9.86
	SSP370 (2081-2100)	100.86	36.70	8.57
	SSP585 (2041-2060)	100.83	36.65	16.53
	SSP585 (2061-2080)	100.85	36.83	20.31
	SSP585 (2081-2100)	100.90	36.85	4.72

G. qinghaiensis	LIG	95.85	28.88	204.21
	LGM	97.79	29.59	106.27
	MH	98.27	30.45	324.18
	Current	97.27	33.24	0
	SSP126 (2041-2060)	96.64	33.38	60.41
	SSP126 (2061-2080)	96.64	33.43	5.12
	SSP126 (2081-2100)	96.64	33.30	15.07
	SSP370 (2041-2060)	96.51	33.51	76.12
	SSP370 (2061-2080)	95.96	33.60	52.31
	SSP370 (2081-2100)	95.97	33.46	15.07
	SSP585 (2041-2060)	96.10	33.25	108.82
	SSP585 (2061-2080)	95.77	33.56	45.96
	SSP585 (2081-2100)	95.76	33.51	5.11

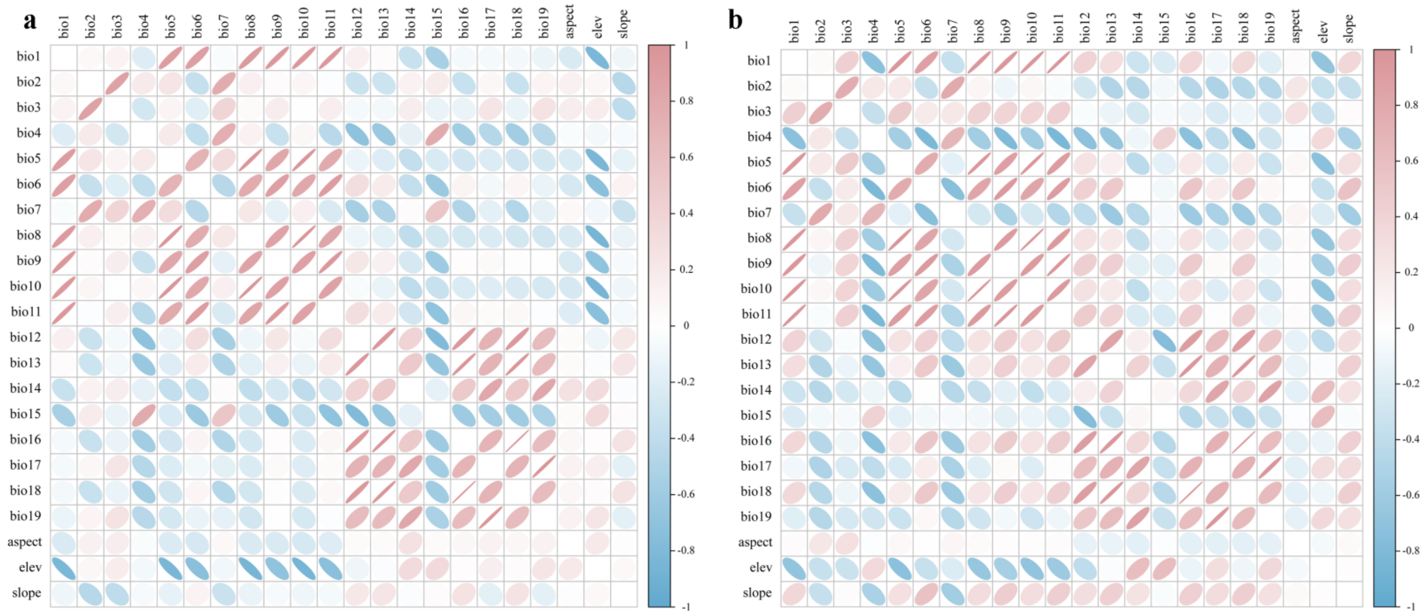

Figure S1 Correlation heatmap of environmental variables.

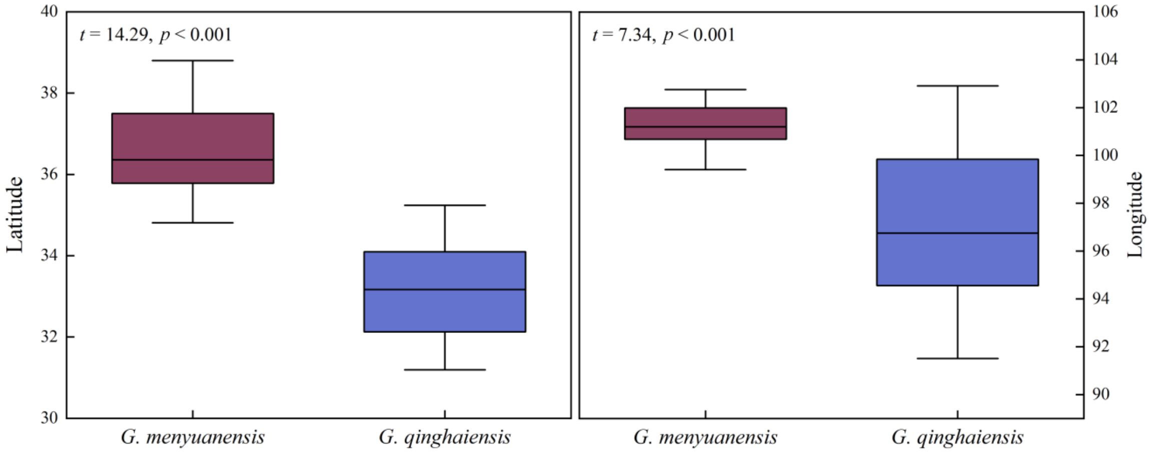

Figure S2 Differences in longitude and latitude between two species.

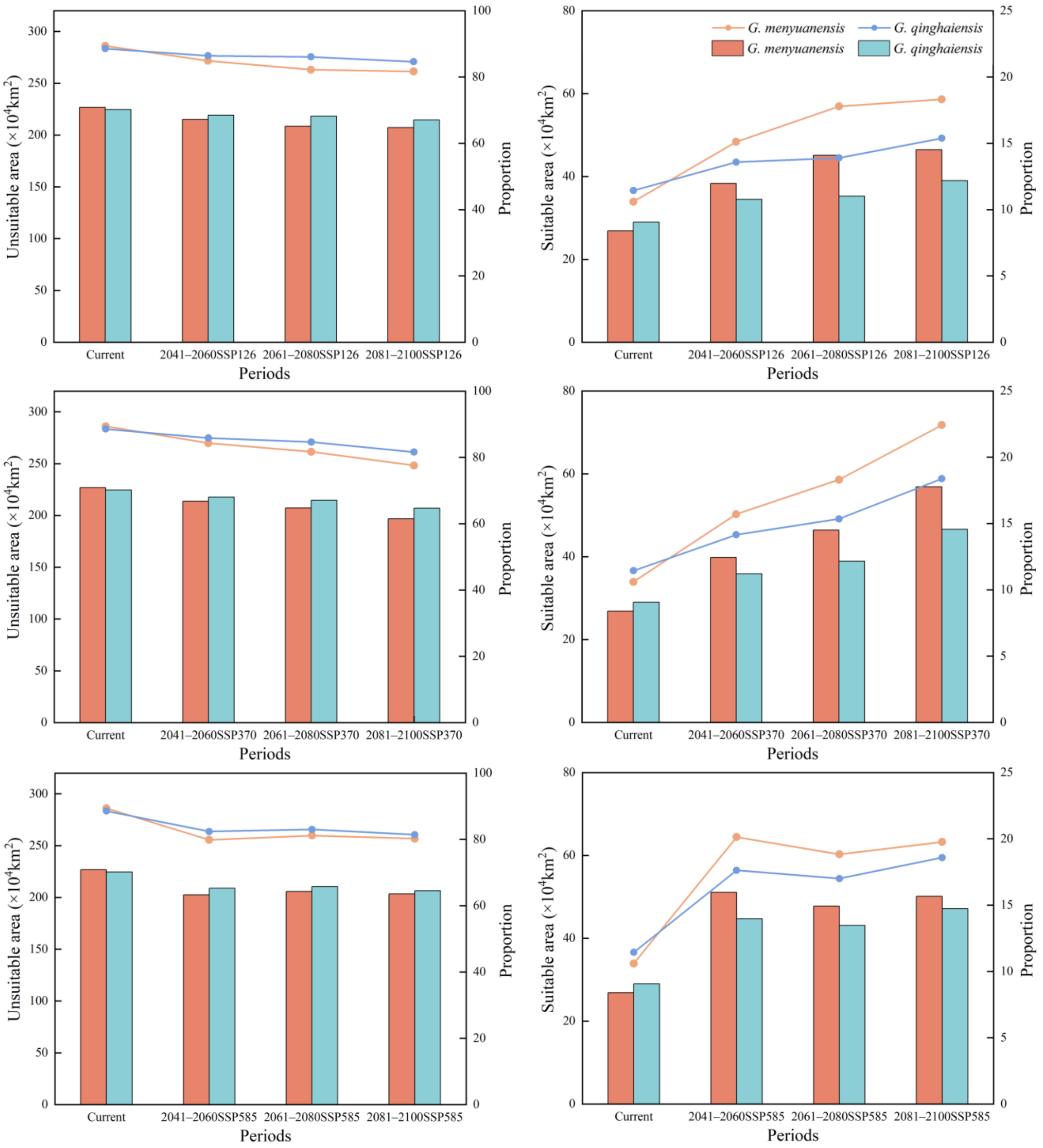

Figure S3 Suitable habitat areas for future period of *Gm* and *Gq*. Bar chart: Area of suitable and unsuitable habitats for *Gm* and *Gq*. Line chart: Proportion of suitable and unsuitable area for *Gm* and *G q*.

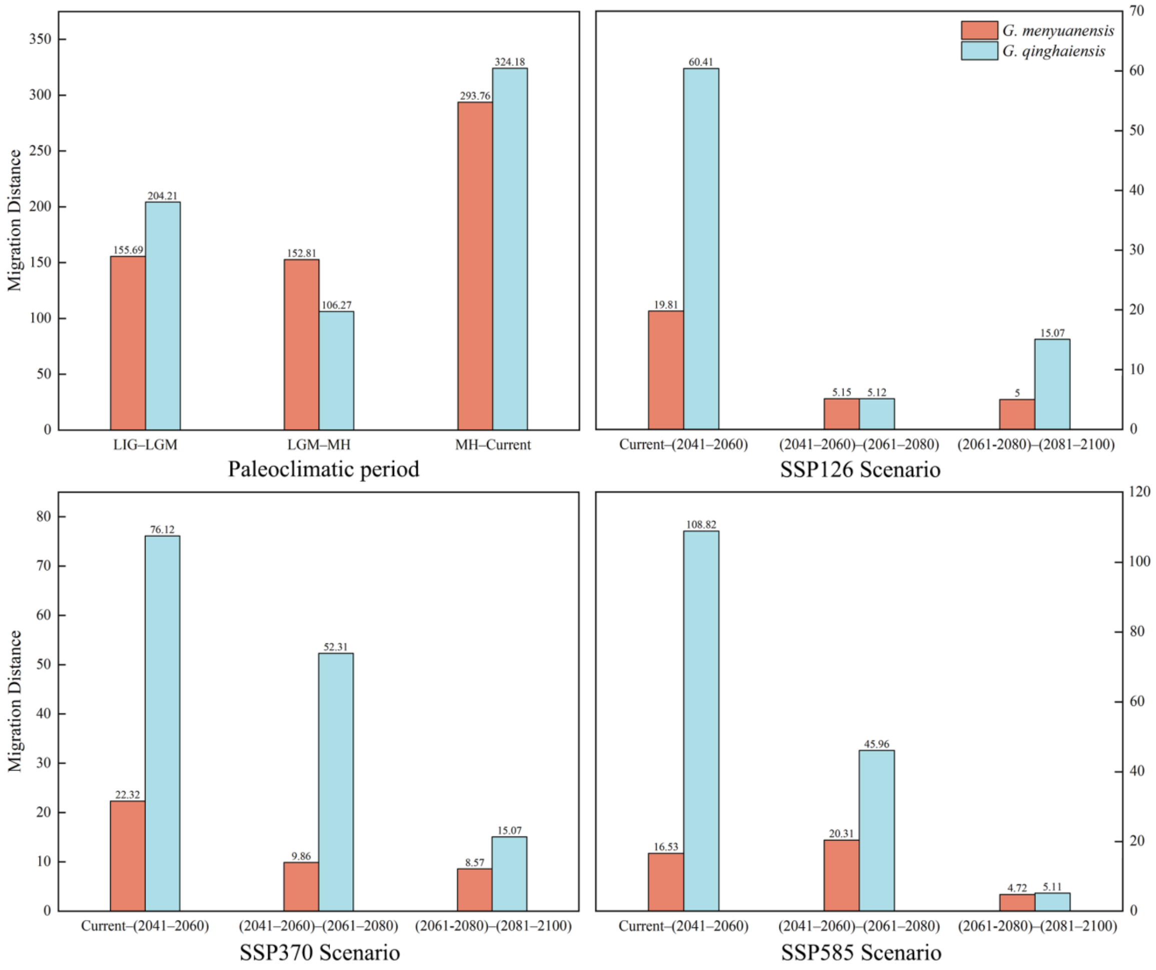

Figure S4 Distribution center migration distance.
